# Associated adverse health outcomes of polypharmacy and potentially inappropriate medications in community-dwelling older adults with diabetes

**DOI:** 10.3389/fphar.2023.1284287

**Published:** 2023-11-16

**Authors:** Lvliang Lu, Shuang Wang, Jiaqi Chen, Yujie Yang, Kai Wang, Jing Zheng, Pi Guo, Yunpeng Cai, Qingying Zhang

**Affiliations:** ^1^ Department of Preventive Medicine, Shantou University Medical College, Shantou, Guangdong, China; ^2^ Shenzhen Health Development Research and Data Management Center, Shenzhen, Guangdong, China; ^3^ Shenzhen Institutes of Advanced Technology, Chinese Academy of Sciences, Shenzhen, Guangdong, China

**Keywords:** polypharmacy, potentially inappropriate medications, adverse health outcome, older, diabetes, clinical practice

## Abstract

**Aim:** This study aimed to identify the association of chronic polypharmacy and potentially inappropriate medications (PIMs) with adverse health outcomes (AHOs) in community-dwelling older adults with diabetes in China.

**Methods:** A 2-year retrospective cohort study was conducted using 11,829 community-followed older adults with diabetes and medical records from 83 hospitals and 702 primary care centers in Shenzhen, China. Chronic polypharmacy and PIMs were identified from prescription records using Beers’ criteria, and their associated AHO was analyzed using multivariable logistic regression analysis.

**Results:** The prevalence of chronic polypharmacy and at least one PIM exposure was 46.37% and 55.09%, respectively. The top five PIMs were diuretics, benzodiazepines, first-generation antihistamines, sulfonylureas, and insulin (sliding scale). Chronic polypharmacy was positively associated with all-cause hospital admission, admission for coronary heart disease, admission for stroke, admission for dementia, and emergency department visits. Exposure to PIMs was positively associated with all-cause hospital admission, admission for heart failure (PIMs ≥2), admission for stroke (PIMs ≥3), emergency department visits, bone fracture, constipation, and diarrhea.

**Conclusion:** Chronic polypharmacy and PIMs were prevalent in older adults with diabetes in Chinese communities. Iatrogenic exposure to chronic polypharmacy and PIMs is associated with a higher incidence of different AHOs. This observational evidence highlights the necessity of patient-centered medication reviews for chronic polypharmacy and PIMs use in older patients with diabetes in primary care facilities in China and draws attention to the caution of polypharmacy, especially PIM use in older adults with diabetes in clinical practice.

## 1 Introduction

According to research conducted in 138 countries with 255 high-quality data sources, China has the highest prevalence of diabetes among people aged over 65 years in the world with 34.1 million patients, accounting for 25.1% of the 135.6 million older adults with diabetes (Sinclair et al., 2020).

Older adults with diabetes often have at least one other chronic disease, such as hypertension, hyperlipidemia, cardiovascular disease, cerebrovascular disease, chronic liver disease, tumors, or chronic respiratory diseases (Wang et al., 2020; Federation, 2022; Ioakeim-Skoufa et al., 2022). Polypharmacy—the use of multiple medications to treat multiple chronic health conditions—is common in older adults with diabetes when clinicians prescribe medications according to the clinical practice guidelines for each chronic comorbidity (Qato et al., 2008; Su et al., 2020; Remelli et al., 2022). According to a systematic review of 173,838 participants, the pooled prevalence of polypharmacy in older patients with type 2 diabetes was 64% (Remelli et al., 2022). In our previous study conducted in outpatient departments in 52 hospitals in Shenzhen, China, we found that the chronic polypharmacy exposure rate ranged from 51% to 55% (Lu et al., 2022).

The risk of potentially inappropriate medication (PIM) exposure in older adults increases by 5.2% with each additional medication added to their medication list (Miller et al., 2017). PIMs are medications that should be avoided in older adults due to the risk of adverse reactions or insufficient evidence of their benefits, especially when safer and equally or more effective therapeutic alternatives are available for the elderly population (BtAGSBCUE, 2019). A meta-analysis of observational studies published between 2002 and 2019 found that the pooled prevalence of PIMs among adults aged 65 years or older in primary care was 33.3% (Liew et al., 2020). In Chinese communities, the prevalence of PIMs ranged from 35.0% to 38.1% (Huang et al., 2020; Li et al., 2021; Su et al., 2022; Tian et al., 2022).

Studies conducted in the Netherlands, Canada, and the United States reported that the prevalence of PIM exposure in older adults with diabetes was 24.9%, 56.1%, and 39.9%, respectively (Gagnon et al., 2020; Nightingale et al., 2021; Oktora et al., 2021). The types and distribution of PIM exposure in older adults with diabetes differed from those without diabetes, as did the amount of medication taken by patients (Gagnon et al., 2020). In our previous study conducted in Shenzhen, China, we found that the prevalence of PIMs in older adults with diabetes ranged from 42% to 45% in outpatient settings (Lu et al., 2022).

Polypharmacy and PIMs have been found to be associated with the incidence of adverse health outcomes (AHO) in older patients, which might be related to drug–drug interactions, side effects of drugs, and reduced physiological functions in older adults, including syncope, dizziness, pain, and emergency department visits (Lohman et al., 2017; Wallace et al., 2017; Davies et al., 2020; Liew et al., 2020; Delgado et al., 2021; Su et al., 2022). However, few studies have examined the patterns of multimorbidity in patients, which are crucial for understanding the iatrogenic exposure to chronic polypharmacy and PIMs, as well as the incidence of AHO (Davies et al., 2020). Our previous research highlighted that the probabilities of exposure and ranking of PIMs in older adults with diabetes, combined with different comorbidities in outpatient visits, were not consistent with chronic polypharmacy (Lu et al., 2022). Considering the significant impact of associated AHO on the health of older adults with diabetes, addressing the issue of chronic polypharmacy and PIMs in older adults with diabetes is of utmost importance in health and drug management (Lohman et al., 2017; Wallace et al., 2017; Davies et al., 2020; Delgado et al., 2021; Su et al., 2022).

It is crucial to study the association between AHO and chronic polypharmacy and PIMs in older adults with diabetes simultaneously. Most studies evaluating the associated AHO of polypharmacy and PIMs were conducted outside China, with implications for different healthcare systems. In this study, we aim to answer two major questions. First, what is the prevalence of chronic polypharmacy and PIMs in older adults with diabetes in the Chinese community? Second, are chronic polypharmacy and PIMs associated with AHO, and is there a dose–response relationship in older adults with diabetes?

## 2 Methods

### 2.1 Data source and study population

This 2-year retrospective cohort study which was accomplished under the guidance of the STROBE checklist used the data on follow-up records of 92,166 diabetic patients registered by community health service centers from the Shenzhen Health Development Research and Data Management Center Database (SHDRDMCD) (Supplementary Table S1). SHDRDMCD also includes medical records of 83 hospitals and 702 primary care centers from 2014 to 2017 in Shenzhen, China. These medical records could entirely reflect each registered patient’s medical institution visits from 2014 to 2017 in Shenzhen, China. Both the diagnostic code and diagnosis name were used for the accurate definition of chronic diseases. The drug name and its unique drug code, frequency, days, and route of administration were combined to embody the prescribed medication. With follow-up records of 92,166 diabetic patients from community health centers and medical records of 83 hospitals and 702 primary care centers, this study could reconstruct the diagnosis and treatment track of older adults with diabetes in Shenzhen, China. An anonymous and standardized medical database was created by assigning a unique identification number to each patient. All the data were checked and imported into the Oracle database by professional platform administrators and medical staff under the supervision of the Shenzhen Municipal Health Commission. According to article No. 32 of the Declaration of Helsinki, the database was approved for research by the Review Committee of the Shenzhen Institute of Advanced Technology, Chinese Academy of Sciences (No. SIAT-IRB-151115- H0084).

The inclusion criteria were confirmed and documented type I and type II diabetic patients who were followed up in 702 community health centers in Shenzhen, China. The included people were aged 65 years or older before 1 January 2015. Older adults with diabetes had two or more medical institution visits and were prescribed at least one medication per visit between the beginning of the cohort (first medical institution visit in 2015) and the end of the cohort (outcomes were observed or 2 years after the start of follow-up). The exclusion criteria were patients who were only prescribed traditional Chinese medicine or Chinese patent medicine at each medical institution visit.

### 2.2 Polypharmacy definition

None, moderate, and severe polypharmacy were defined as the use of 0–4, 5–9, and ≥10 chronically used drugs, respectively (Masnoon et al., 2017; Organization). Only medication that was used for a long term (defined by the use of drugs for more than 90 days or at least once a month) was investigated. The third level of the Anatomical Therapeutic Chemical (ATC) code was used to calculate the number of different chronic drugs used. Therefore, the use of chronically used drugs with different substances in the same pharmacological subgroup could be considered as the use of one chronically used drug, such as angiotensin receptor blockers (ATC code = C09C). Drugs prescribed for topical treatment, surgical dressing, contrast media, radiopharmaceuticals, and general nutrients, as well as drugs without ATC codes, such as Chinese patent medications, were excluded from the evaluation of polypharmacy. Drug combinations with different third-level ATC codes were defined as two drugs. The definition of chronic polypharmacy exposure was estimated during the inclusion period.

### 2.3 Potentially inappropriate medication definition

The American Geriatrics Society 2019 Beers Criteria were used to identify PIM exposure (BtAGSBCUE, 2019). Some PIM items could not be evaluated in older adults with diabetes in China for the following reasons. First, some laboratory data that were critical for PIM evaluation were lacking. Second, SHDRDMCD has an inconsistent presentation of drug doses, such as one capsule or one tablet. Third, the concomitant use of drugs in patients cannot be located. Therefore, PIM categories IV and V and parts of categories I, II, and III (Supplementary Table S2) of the Beers Criteria could not be assessed in this study. Some PIM items were not available in the Chinese healthcare system. We formulated a list of 42 PIM items in accordance with the characteristics of the Chinese healthcare system and SHDRDMCD to identify the exposure of PIMs in Chinese communities (Supplementary Table S2). The list also includes the corresponding notes for inclusion and reasons for exclusion of PIM items. We specified the patients’ disease or syndrome by means of the 10th edition of the International Classification of Disease (ICD-10) codes, which required the category II PIM item assessment. PIMs in older adults with diabetes in Chinese communities were stratified into four levels: 0, 1, 2, and 3 or more PIM exposures. The definition of PIM exposure was estimated during the inclusion period.

### 2.4 Comorbidity definition

Patterns of multimorbidity must be associated with the emergence of chronic polypharmacy and PIMs, as well as their associated AHO in older adults with diabetes. The ATC drug code categories and patterns of multimorbidity in Chinese elderly individuals were consulted for the definition and selection of the investigated chronic disease (Wang et al., 2020; Han et al., 2022). Finally, 10 chronic diseases were selected for adjustment. The corresponding ICD-10 codes are attached to Supplementary Table S3. The earliest diagnoses and ICD-10 codes in SHDRDMCD before the start of the follow-up were accepted for the definition of chronic comorbidities, except for tumors, which were required within 5 years earlier. Comorbidities were presented with or without the chosen disease in addition to diabetes.

### 2.5 Covariates

Age, systolic blood pressure, diastolic blood pressure, body mass index, fasting blood glucose, 2-h postprandial glucose, and glycosylated hemoglobin were collected as continuous variables prior to the beginning of the cohort. Age was stratified into four groups of 65–69, 70–74, 75–79, and ≥80 years, and BMI was stratified into four groups of <18.5, 18.5–24.0, 24.0–28.0, and ≥28.0 kg/m^2^. The complications of diabetes were presented with or without the terms of the diagnoses and corresponding ICD-10 codes in SHDRDMCD.

### 2.6 Associated adverse health outcomes

The AHO included all-cause hospital admission; hospital admission for coronary heart disease, stroke, dementia, and heart failure; emergency department visits; bone fractures; constipation; and diarrhea in this study. The follow-up ended with the first occurrence of AHO or lasted 2 years after the beginning of the cohort. Finally, AHO was collected as a dichotomous variable for analysis.

### 2.7 Statistical analysis

The prevalence of chronic polypharmacy and PIMs among older adults with diabetes is presented as percentages with 95% CIs. Chi-squared tests were used to compare the categorical variables of the baseline characteristics. The analysis of variance and the Kruskal‒Wallis test were used for continuous variables with and without normal distribution, respectively.

Patients who had a diagnosis or the etiology of hospital admission same as AHO within 6 months prior to the start of the cohort were excluded from the statistical analysis. Univariable and multivariable logistic regression analyses were performed to assess the risk of AHO in older adults with diabetes who were exposed to chronic polypharmacy and PIMs (no exposure to chronic polypharmacy or PIMs as a reference). Multivariable logistic regression analyses were performed for adjusting by including all variables listed in the baseline characteristics. The classification and regression tree methods were used to perform multiple interpolations for missing values of variables. The sensitivity analysis which was used to compare the results of logistic regression before and after multiple interpolations is provided in Supplementary Tables S4, S5. A two-sided *α* = 0.05 was considered statistically significant. The generalized variance-inflation factors (GVIFs) were applied for a multicollinearity assessment of all variables included for adjusted logistic regression. A GVIF value >10 was considered a strong multicollinearity. All analyses were performed in R 4.1.2 (R Development Core Team).

## 3 Results

### 3.1 Baseline characteristics

**FIGURE 1:**
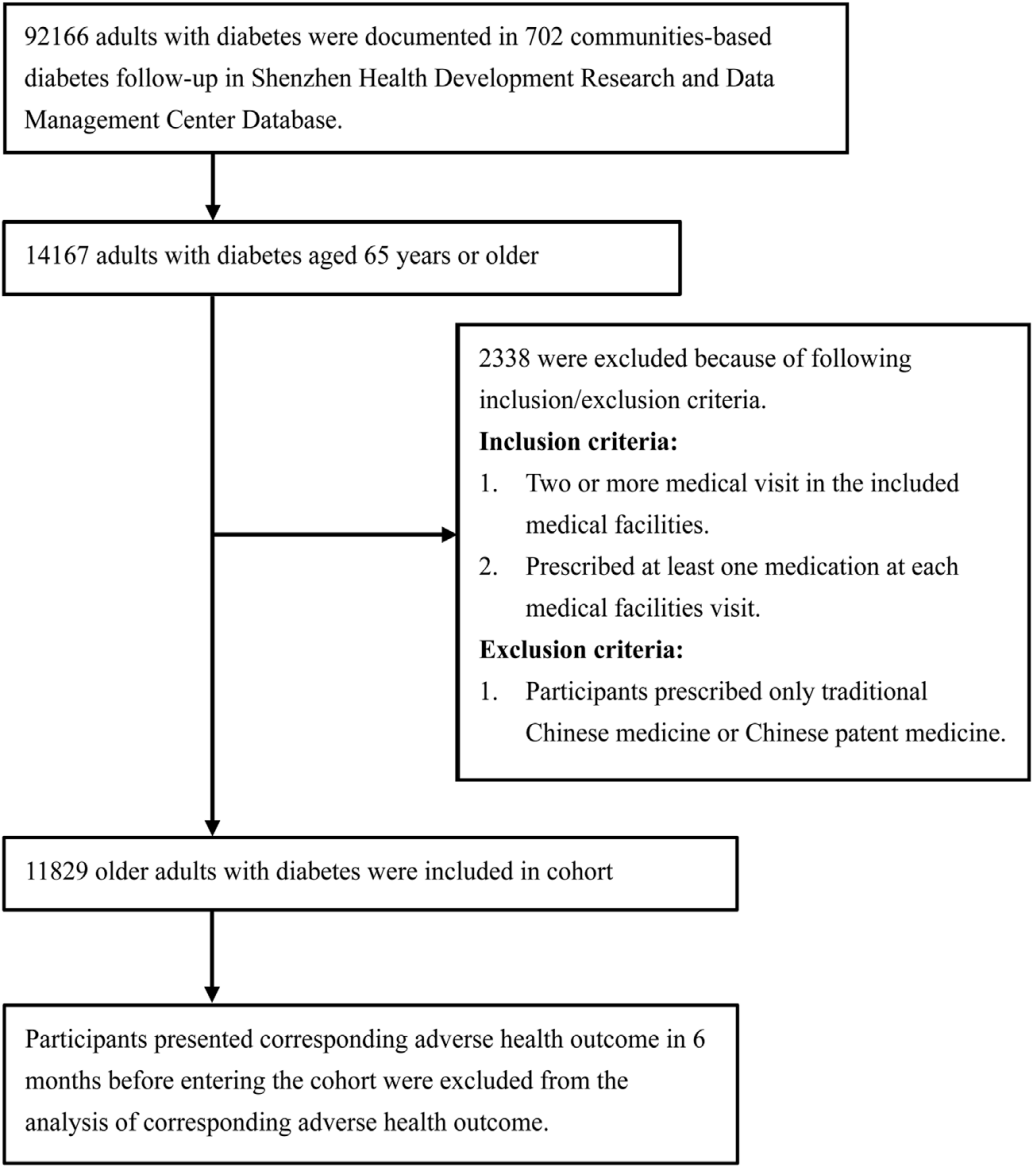
Flowchart of participant selection.

A total of 11,829 community-followed older adults with diabetes were enrolled in this study, with 53.54% being women (Figure 1). The baseline characteristics of the included population are shown in Table 1. The number of patients who experienced all-cause hospital admission was 4,142 (35.02%), with 784 (6.63%) for hospital admission for coronary heart disease, 677 (5.72%) for hospital admission for stroke, 134 (1.13%) for hospital admission for dementia, 67 (0.57%) for hospital admission for heart failure, 3,110 (26.29%) for emergency department visits, 580 (4.90%) for bone fracture, 932 (7.88%) for constipation, and 167 (1.41%) for diarrhea.

**TABLE 1:** Baseline characteristics of included 11,829 community-followed older adults with diabetes.

	Potentially inappropriate medications					Polypharmacy			
	0	1	2	≥3	*p*	None	Moderate	Severe	*p*
**Age, y (n = 11,829)**					<0.001				<0.001
65–69	2,106 (17.80)	1,223 (10.34)	577 (4.88)	453 (3.83)		2,541 (21.48)	1,530 (12.93)	288 (2.43)	
70–74	1,371 (11.59)	887 (7.50)	405 (3.42)	367 (3.10)		1,627 (13.75)	1,132 (9.57)	271 (2.29)	
75–79	1,002 (8.47)	664 (5.61)	369 (3.12)	327 (2.76)		1,170 (9.89)	908 (7.68)	284 (2.40)	
≥80	833 (7.04)	543 (4.59)	310 (2.62)	392 (3.31)		1,006 (8.50)	741 (6.26)	331 (2.80)	
**Gender (n = 11,829)**					0.039				<0.001
Male	2,502 (21.15)	1,548 (13.09)	774 (6.54)	672 (5.68)		2,772 (23.43)	2,083 (17.61)	641 (5.42)	
Female	2,810 (23.76)	1,769 (14.95)	887 (7.50)	867 (7.33)		3,572 (30.20)	2,228 (18.84)	533 (4.51)	
**SBP (n = 11,584)**	129.93 (11.17)	129.57 (10.44)	129.75 (10.46)	129.51 (10.06)	0.165	129.59 (11.26)	130.03 (10.04)	129.57 (10.20)	0.264
**DBP (n = 11,582)**	78.23 (7.01)	77.94 (6.84)	77.74 (6.68)	77.77 (6.91)	<0.001	78.02 (7.06)	78.04 (6.68)	77.69 (6.93)	0.053
**BMI (n = 11,616)**					0.299				0.003
<18.5	105 (0.90)	55 (0.47)	25 (0.22)	29 (0.25)		135 (1.14)	57 (0.48)	22 (0.49)	
18.5–24	3,300 (28.41)	2,118 (18.23)	1,013 (8.72)	923 (7.95)		4,007 (33.87)	2,642 (22.33)	705 (5.96)	
24–28	1,529 (13.16)	908 (7.82)	518 (4.46)	464 (3.99)		1,774 (15.00)	1,287 (10.88)	358 (3.03)	
≥28	300 (2.58)	167 (1.44)	71 (0.61)	91 (0.78)		328 (2.77)	246 (2.08)	55 (0.46)	
**FPG (n = 11,739)**	7.2 (1.82)	7.11 (1.75)	7.1 (1.78)	7.07 (1.73)	0.004	7.21 (1.84)	7.06 (1.69)	7.08 (1.79)	<0.001
**2 h-PBG (n = 10,014)**	9.44 (2.30)	9.41 (2.15)	9.26 (2.13)	9.26 (2.13)	0.001	9.47 (2.25)	9.30 (2.14)	9.24 (2.32)	<0.001
**HbA1c (n = 9,601)**	6.49 (1.45)	6.48 (1.42)	6.52 (1.48)	6.40 (1.45)	0.04	6.50 (1.49)	6.46 (1.42)	6.42 (1.34)	0.037
Complication, n (%)									
DPN					<0.001				<0.001
With (n = 689)	183 (1.55)	212 (1.79)	131 (1.11)	163 (1.38)		168 (1.42)	364 (3.08)	157 (1.33)	
Without (n = 11,140)	5,129 (43.36)	3,105 (26.25)	1,530 (12.93)	1,376 (11.63)		6,176 (52.21)	3,947 (33.37)	1,017 (8.60)	
DKD					<0.001				<0.001
With (n = 441)	87 (0.74)	125 (1.06)	92 (0.78)	137 (1.16)		87 (0.74)	203 (1.72)	151 (1.28)	
Without (n = 11,388)	5,225 (44.17)	3,192 (26.98)	1,569 (13.26)	1,402 (11.85)		6,257 (52.90)	4,108 (34.73)	1,023 (8.65)	
DR					<0.001				<0.001
With (n = 354)	120 (1.01)	93 (0.79)	61 (0.52)	80 (0.68)		104 (0.88)	164 (1.39)	86 (0.73)	
Without (n = 11,475)	5,192 (43.89)	3,224 (27.25)	1,600 (13.53)	1,459 (12.33)		6,240 (52.75)	4,147 (35.06)	1,088 (9.20)	
Comorbidity, n (%)									
CRD					<0.001				<0.001
With (n = 1,095)	303 (2.56)	260 (2.20)	223 (1.89)	309 (2.61)		348 (2.94)	475 (4.02)	272 (2.30)	
Without (n = 10,734)	5,009 (42.35)	3,057 (25.84)	1,438 (12.16)	1,230 (10.40)		5,996 (50.69)	3,836 (32.43)	902 (7.63)	
OARA					<0.001				<0.001
With (n = 2,522)	666 (5.63)	747 (6.31)	498 (4.21)	611 (5.17)		892 (7.54)	1,168 (9.87)	462 (3.91)	
Without (n = 9,307)	4,646 (39.28)	2,570 (21.73)	1,163 (9.83)	928 (7.85)		5,452 (46.09)	3,143 (26.57)	712 (6.02)	
CLD					<0.001				<0.001
With (n = 369)	115 (0.97)	85 (0.72)	83 (0.70)	86 (0.73)		114 (0.96)	165 (1.39)	90 (0.76)	
Without (n = 11,460)	5,197 (43.93)	3,232 (27.32)	1,578 (13.34)	1,453 (12.28)		6,230 (52.67)	4,146 (35.05)	1,084 (9.16)	
Hypertension					<0.001				<0.001
With (n = 8,363)	3,246 (27.44)	2,437 (20.60)	1,335 (11.29)	1,345 (11.37)		3,379 (28.57)	3,843 (32.49)	1,141 (9.65)	
Without (n = 3,466)	2,066 (17.47)	880 (7.44)	326 (2.76)	194 (1.64)		2,965 (25.07)	468 (3.96)	33 (0.28)	
Hyperlipidemia					<0.001				<0.001
With (n = 3,729)	1,286 (10.87)	1,138 (9.62)	629 (5.32)	676 (5.71)		956 (8.08)	2,035 (17.20)	738 (6.24)	
Without (n = 8,100)	4,026 (34.03)	2,179 (18.42)	1,032 (8.72)	863 (7.30)		5,388 (45.55)	2,276 (19.24)	436 (3.69)	
CBD					<0.001				<0.001
With (n = 1,626)	484 (4.09)	450 (3.80)	308 (2.60)	384 (3.25)		395 (3.34)	827 (6.99)	404 (3.42)	
Without (n = 10,203)	4,828 (40.81)	2,867 (24.24)	1,353 (11.44)	1,155 (9.76)		5,949 (50.29)	3,484 (29.45)	770 (6.51)	
CKD					<0.001				<0.001
With (n = 667)	144 (1.22)	168 (1.42)	129 (1.09)	226 (1.91)		135 (1.14)	292 (2.47)	240 (2.03)	
Without (n = 11,162)	5,168 (43.69)	3,149 (26.62)	1,532 (12.95)	1,313 (11.10)		6,209 (52.49)	4,019 (33.98)	934 (7.90)	
CGD					<0.001				<0.001
With (n = 117)	20 (0.17)	23 (0.19)	29 (0.25)	45 (0.38)		24 (0.20)	43 (0.36)	50 (0.42)	
Without (n = 11,712)	5,292 (44.74)	3,294 (27.85)	1,632 (13.80)	1,494 (12.63)		6,320 (53.43)	4,268 (36.08)	1,124 (9.50)	
CVD					<0.001				<0.001
With (n = 4,510)	1,460 (12.34)	1,324 (11.19)	795 (6.72)	931 (7.87)		1,261 (10.66)	2,299 (19.44)	950 (8.03)	
Without (n = 7,319)	3,852 (32.56)	1,993 (16.85)	866 (7.32)	608 (5.14)		5,083 (42.97)	2,012 (17.01)	224 (1.89)	
Tumor					<0.001				<0.001
With (n = 393)	113 (0.96)	113 (0.96)	80 (0.68)	87 (0.74)		140 (1.18)	183 (1.55)	70 (0.59)	
Without (n = 11,436)	5,199 (43.95)	3,204 (27.09)	1,581 (13.37)	1,452 (12.27)		6,204 (52.45)	4,128 (34.90)	1,104 (9.33)	

Notes: none, moderate, and severe polypharmacy were defined as 0–4, 5–9, and ≥10 chronic used drugs, respectively. SBP, systolic blood pressure; DBP, diastolic blood pressure; BMI, body mass index; FBG, fasting plasma glucose; 2 h-PBG, 2-h postprandial blood glucose; HbA1c = glycated hemoglobin; DPN, diabetic peripheral disease; DKD, diabetic kidney disease; DR, diabetic retinal; CRD, chronic respiratory disease; OARA, osteoarthritis and rheumatoid arthritis; CLD, chronic liver disease; CBD, cerebrovascular disease; CKD, chronic kidney disease; CGD, chronic gastrointestinal disease; CVD, cardiovascular disease. SBP, DBP, FPG, 2 h-PBG, and HbA1c were presented as mean (SD), and the rest of indicators were presented as n (%).

### 3.2 Prevalence of polypharmacy and associated AHO

The prevalence of chronic polypharmacy in this cohort was 46.37% (95% CI: 45.55–47.19), with 36.45% (35.72–37.18) moderate polypharmacy and 9.93% (9.55–10.31) severe polypharmacy.

The univariable analysis revealed that chronic polypharmacy was associated with the incidence of any AHO. A multicollinearity test of the baseline characteristics of older adults with diabetes showed that none of the GVIF values were greater than 10, suggesting no significant multicollinearity among the variables (Supplementary Tables S6–S14). The results of the sensitivity test indicated that the effect size was stable before and after multiple interpolations (Supplementary Table S4). Multivariable logistic regression analysis revealed that chronic polypharmacy had a positive correlative dose‒response relationship with the incidence of AHO (all-cause hospital admission: moderate: a OR = 1.95, 95% CI 1.76–2.17; severe: 2.86, 2.38–3.43; hospital admission for coronary heart disease: moderate: 2.00, 1.59–2.53; severe: 5.76, 4.28–7.78; stroke: moderate: 2.05, 1.62–2.60; severe: 2.48, 1.78–3.47; dementia: moderate: 1.80, 1.08–3.05; severe: 3.61, 1.84–7.15; and emergency department visit: moderate: 1.38, 1.23–1.55; severe: 1.75, 1.45–2.10; Table 1). However, there was no relevance between chronic polypharmacy and the occurrence of hospital admission for heart failure, bone fracture, constipation, and diarrhea in older adults with diabetes (Table 2).

**TABLE 2:** Associated adverse health outcomes of polypharmacy in community-followed older adults with diabetes.

	Univariate analyses			Adjusted logistic regression		
	OR	95% CI	*p*	aOR	95% CI	*p*
All-cause hospital admission (n = 4,142)						
Moderate	2.91	(2.67, 3.17)	<0.001	1.95	(1.76, 2.17)	<0.001
Severe	7.11	(6.21, 8.14)	<0.001	2.86	(2.38, 3.43)	<0.001
Hospital admission for coronary heart disease (n = 784)						
Moderate	3.47	(2.85, 4.24)	<0.001	2.00	(1.59, 2.53)	<0.001
Severe	14.77	(12.01, 18.24)	<0.001	5.76	(4.28, 7.78)	<0.001
Hospital admission for stroke (n = 677)						
Moderate	3.27	(2.70, 3.96)	<0.001	2.05	(1.62, 2.60)	<0.001
Severe	6.69	(5.35, 8.37)	<0.001	2.48	(1.78, 3.47)	<0.001
Hospital admission for dementia (n = 134)						
Moderate	2.56	(1.68, 3.98)	<0.001	1.80	(1.08, 3.05)	0.025
Severe	7.45	(4.74, 11.83)	<0.001	3.61	(1.84, 7.15)	<0.001
Hospital admission for heart failure (n = 67)						
Moderate	1.77	(0.89, 3.56)	0.103	0.69	(0.30, 1.58)	0.369
Severe	12.59	(6.97, 23.86)	<0.001	1.59	(0.62, 4.25)	0.343
Emergency department admission (n = 3,110)						
Moderate	2.55	(2.33, 2.80)	<0.001	1.38	(1.23, 1.55)	<0.001
Severe	6.58	(5.76, 7.52)	<0.001	1.75	(1.45, 2.10)	<0.001
Bone fracture (n = 580)						
Moderate	1.48	(1.22, 1.78)	<0.001	0.93	(0.74, 1.17)	0.539
Severe	3.11	(2.47, 3.90)	<0.001	1.13	(0.80, 1.59)	0.484
Constipation (n = 932)						
Moderate	2.11	(1.81, 2.45)	<0.001	1.19	(0.97, 1.45)	0.119
Severe	4.02	(3.23, 4.86)	<0.001	1.22	(0.92, 1.61)	0.166
Diarrhea (n = 167)						
Moderate	1.61	(1.12, 2.30)	0.009	0.80	(0.52, 1.24)	0.32
Severe	4.15	(2.78, 6.15)	<0.001	0.91	(0.49, 1.67)	0.765

Abbreviations: OR, odds ratio; aOR, adjusted odds ratio; 95% CI, 95% confidence interval. Multivariate adjusted logistic regression was achieved by including every variable presented in baseline characteristics table.

### 3.3 Prevalence of PIMs and associated AHO

The prevalence of at least one PIM exposure in this cohort was 55.09% (95% CI: 54.19–55.99). Among them, 28.04% (27.40–28.68), 14.04% (13.59–14.49), and 12.01% (11.59–12.43) of patients had one, two, and three or more PIM exposures, respectively. When we classified PIMs into drug classes, the top five most commonly used PIMs were diuretics (15.04%), benzodiazepines (13.63%), first-generation antihistamines (11.65%), sulfonylureas (5.86%), and insulin (sliding scale) (5.09%). The specific PIMs identified in this cohort are listed in Supplementary Table S15.

A univariable logistic regression showed that PIMs were associated with any AHO. The sensitivity test presented stable effect sizes (Supplementary Table S5). Multivariable logistic regression revealed that PIMs were positively associated with the incidence of AHO, with a dose‒response relationship (bone fracture: 1 PIM: aOR = 1.82, 95% CI 1.44–2.31; 2 PIMs: 2.13, 1.61–2.81; 3 or more PIMs: 2.73, 2.03–3.67; constipation: 1 PIM: 1.18, 0.97–1.43; 2 PIMs: 1.43, 1.14–1.78; 3 or more PIMs: 2.00, 1.59–2.52; diarrhea: 1 PIM: 1.12, 0.69–1.80; 2 PIMs: 2.70, 1.68–4.34; 3 or more PIMs: 2.78, 1.64–4.73; emergency department visiting: 1 PIM: 1.66, 1.49–1.86; 2 PIMs: 1.99, 1.73–2.28; 3 or more PIMs: 2.92, 2.50–3.40; and all-cause hospital admission: 1 PIM: 1.22, 1.10–1.35; 2 PIMs: 1.38, 1.21–1.57; 3 or more PIMs: 1.62, 1.39–1.87; Table 3). However, PIMs had no impact on the incidence of hospital admission for coronary heart disease and dementia. An increased number of PIM exposures was related to the occurrence of hospital admission for stroke (3 or more PIMs: OR = 1.35, 95% CI 1.03–1.77) and heart failure (2 PIMs: 3.17, 1.13–9.37; 3 or more PIMs: 6.97, 2.60–20.48) (Table 3).

**TABLE 3:** Associated adverse health outcomes of potentially inappropriate medications in community-followed older adults with diabetes.

	Univariate analyses			Adjusted logistic regression		
	OR	95% CI	*p*	aOR	95% CI	*p*
All-cause hospital admission (n = 4,142)						
1	1.65	(1.50, 1.81)	<0.001	1.22	(1.10, 1.35)	<0.001
2	2.45	(2.18, 2.75)	<0.001	1.38	(1.21, 1.57)	<0.001
≥3	4.19	(3.72, 4.72)	<0.001	1.62	(1.39, 1.87)	<0.001
Hospital admission for coronary heart disease (n = 784)						
1	2.03	(1.66, 2.48)	<0.001	1.28	(0.96, 1.66)	0.123
2	2.66	(2.13, 3.33)	<0.001	1.11	(0.86, 1.43)	0.411
≥3	4.90	(4.00, 6.01)	<0.001	1.04	(0.80, 1.36)	0.758
Hospital admission for stroke (n = 677)						
1	1.54	(1.25, 1.90)	<0.001	1.06	(0.85, 1.33)	0.605
2	2.19	(1.73, 2.76)	<0.001	1.16	(0.89, 1.51)	0.266
≥3	3.49	(2.82, 4.31)	<0.001	1.35	(1.03, 1.77)	0.033
Hospital admission for dementia (n = 134)						
1	2.28	(1.42, 3.71)	<0.001	1.69	(0.93, 2.91)	0.141
2	3.12	(1.85, 5.27)	<0.001	1.66	(0.87, 3.01)	0.182
≥3	4.36	(2.67, 7.19)	<0.001	1.40	(0.76, 2.62)	0.283
Hospital admission for heart failure (n = 67)						
1	2.52	(0.99, 6.86)	0.056	2.09	(0.80, 5.84)	0.139
2	5.05	(1.98, 13.74)	<0.001	3.17	(1.13, 9.37)	0.03
≥3	19.19	(9.11, 47.03)	<0.001	6.97	(2.60, 20.48)	<0.001
Emergency department admission (n = 3,110)						
1	2.09	(1.88, 2.33)	<0.001	1.66	(1.49, 1.86)	<0.001
2	3.10	(2.73, 3.51)	<0.001	1.99	(1.73, 2.28)	<0.001
≥3	6.16	(5.43, 6.98)	<0.001	2.92	(2.50, 3.40)	<0.001
Bone fracture (n = 580)						
1	2.04	(1.62, 2.56)	<0.001	1.82	(1.44, 2.31)	<0.001
2	2.61	(2.02, 3.38)	<0.001	2.13	(1.61, 2.81)	<0.001
≥3	4.32	(3.42, 5.47)	<0.001	2.73	(2.03, 3.67)	<0.001
Constipation (n = 932)						
1	1.55	(1.29, 1.85)	<0.001	1.18	(0.97, 1.43)	0.125
2	2.13	(1.74, 2.60)	<0.001	1.43	(1.14, 1.78)	0.002
≥3	3.71	(3.09, 4.46)	<0.001	2.00	(1.59, 2.52)	<0.001
Diarrhea (n = 167)						
1	1.28	(0.80, 2.04)	0.293	1.12	(0.69, 1.80)	0.656
2	3.50	(2.27, 5.42)	<0.001	2.70	(1.68, 4.34)	<0.001
≥3	4.61	(3.05, 7.03)	<0.001	2.78	(1.64, 4.73)	<0.001

Abbreviations: OR, odds ratio; aOR, adjusted odds ratio; 95% CI, 95% confidence interval. Multivariate adjusted logistic regression was achieved by including every variable presented in baseline characteristics table.

## 4 Discussion

This study provides estimates for chronic polypharmacy and PIM prevalence and their associated AHO in a large, representative sample of community-dwelling older adults with diabetes in China. The prevalence of chronic polypharmacy and PIMs in older adults with diabetes in the Chinese community was 46.37% and 55.09%, respectively. Remarkably, after adjusting for patients’ baseline characteristics and three complications of diabetes, as well as ten comorbidities, PIM exposure was associated with the incidence of bone fracture, constipation, diarrhea, emergency department visits, all-cause hospital admissions, and hospital admissions for stroke and heart failure in older adults with diabetes. In contrast to PIM use, chronic polypharmacy was associated with the incidence of all-cause hospital admissions, emergency department visits, and hospital admissions for coronary heart disease, stroke, and dementia.

The prevalence of chronic polypharmacy among older adults with diabetes in Chinese communities was 46.37%, indicating that it was an un-neglected issue in the management of polypharmacy. Only chronically used medication and the third level of the ATC codes that were applied may partly explain the lower prevalence of polypharmacy than in previous studies of polypharmacy prevalence among older adults with diabetes around the world (Remelli et al., 2022). A 5-year repeated cross-sectional study showed that the change in polypharmacy prevalence was smaller in older adults with diabetes than in middle-aged patients, which might be related to the steady type and number of chronic diseases in older adults with diabetes (Oktora et al., 2021). Assessing the prevalence of chronic polypharmacy will be more suitable for older adults with diabetes. We accurately assessed the prevalence of iatrogenic chronic polypharmacy of older adults with diabetes in the Chinese community as 46.37% with the medical records of 83 hospitals and 702 primary care centers documented in SHDMDRCD.

The 55.09% prevalence of PIMs is also an urgent concern in the health and medication management of older adults with diabetes in China. With SHDRDMCD and 39 out of 42 PIM items in the 2019 Beers Criteria, this study provides a complete representation of the iatrogenic PIM exposure rate in community-dwelling older adults with diabetes in Shenzhen, China. The prevalence of PIMs in this study is significantly higher than those of existing cross-sectional studies in the Netherlands (24.9%) and the United States (39.9%) but is similar to the retrospective cohort study conducted in Canada (56.1%) (Gagnon et al., 2020; Nightingale et al., 2021; Oktora et al., 2021). It must be related to the fact that only 24 PIM items of the 2015 Beers Criteria were evaluated in 60 community pharmacies in the Netherlands, and 40 PIM items of the 2019 Beers Criteria were evaluated in an emergency department in the United States. Similar to this study, all accessible healthcare facilities were evaluated in a retrospective cohort study in Quebec, Canada. This indicates that iatrogenic PIM exposure in older adults with diabetes could exceed 50%.

In older adults with diabetes, inappropriate medication was associated with an increased risk of cardiovascular disease, stroke, dementia, gastrointestinal autonomic dysfunction, and osteoporosis (Ling et al., 2000; Cukierman-Yaffe et al., 2009; Cavender et al., 2015; Pan et al., 2019; Gerontology NCo et al., 2021). The health status of older adults with diabetes will be seriously affected once they are hospitalized for coronary heart disease, heart failure, dementia, stroke, bone fracture, constipation, and diarrhea (Cukierman-Yaffe et al., 2009; Cavender et al., 2015; Gilbert and Pratley, 2015; Pan et al., 2019; Gerontology NCo et al., 2021). In response to such a high prevalence of chronic polypharmacy and PIMs in older adults with diabetes, it was critical to investigate whether it would cause a negative impact on the patient’s health.

Importantly, this study simultaneously investigates the relationship between exposure to chronic polypharmacy and PIMs with AHO. A systematic review summarized that evidence of adverse drug events, falls, bone fractures, gastrointestinal symptoms, and circulatory disease in older individuals exposed to polypharmacy was inconsistent or contradictory (Davies et al., 2020). The same phenomenon could be observed in studies concentrating on PIMs (Liew et al., 2020; Weir et al., 2020; Bories et al., 2021). Considering that different probabilities and inconsistent rankings of exposure to chronic polypharmacy and PIMs in older adults with diabetes combined with different chronic comorbidities and increasing exposure to polypharmacy increased the probability of PIM exposure, we presumed that separately exploring the AHO of polypharmacy and PIM exposure would uncover the real evidence (Miller et al., 2017; Lu et al., 2022). To avoid confounding factors, people who suffered from the investigated AHO within 6 months before the start of the follow-up were excluded from the corresponding analysis. Ultimately, with SHDRDMCD, we found different associated AHO between exposure to chronic polypharmacy and PIMs in older adults with diabetes. Compared with chronic polypharmacy, PIMs were associated with more AHO-like bone fractures, constipation, and diarrhea, in older adults with diabetes. This study suggests that more attention should be paid to the substitution or withdrawal of PIMs in older adults with diabetes in clinical practice and drug management to reduce AHO.

Optimization of the drug list concerning chronic polypharmacy and PIMs in clinical practice should pay more attention to the comorbidity of patients and possible AHO. The risk of all-cause hospital admission and emergency department visits, which are commonly explored in existing studies, could not provide specific adverse health impacts of exposure to chronic polypharmacy and PIMs, regardless of the angle of the clinical practitioner or patient. The results of this study could serve as a basis for a drug-list review to avoid excessive blood glucose fluctuations due to drug‒drug interactions and a high risk of bleeding, which might lead to hospital admission for dementia, stroke, and coronary heart disease. For example, repaglinide may enhance and/or prolong the hypoglycemic effect of repaglinide and, thereby, increase the risk of hypoglycemia when combined with clopidogrel, ketoconazole, and angiotensin-converting enzyme inhibitors (Plosker and Figgitt, 2004; Takayama et al., 2021). The combination of acarbose and warfarin will increase the risk of bleeding by increasing the international normalized ratio of prothrombin (Dash et al., 2018). Replacement or withdrawal of drugs by reviewing the possible risk of AHO in older adults with diabetes who were exposed to PIMs was practical. For example, short-acting insulin and sulfonylureas predispose patients to hypoglycemia, which can increase the risk of falling and, thus, fracture (BtAGSBCUE, 2019). Precise indications for possible AHO of chronic polypharmacy and PIMs are important in optimizing treatment.

## 5 Practical implications

This observational study highlights that chronic polypharmacy and PIMs were prevalent in community-dwelling older adults with diabetes. The study’s findings contribute to improving the awareness among primary healthcare workers regarding the AHO of polypharmacy and PIMs use in older adults with diabetes. The quantity of medications and the utilization of PIMs may serve as significant mediators for AHO, making them valuable indicators for primary healthcare workers to periodically review the medication needs of older patients with diabetes.

Patient-centered medication review was required in disease management for older adults with diabetes regarding chronic polypharmacy and PIM use in primary care facilities in China. Many specialty clinics may add new drugs to address specific issues without fully considering the comprehensive health status of older diabetes patients and their existing medication regimens for other chronic conditions. Since the widespread adoption of disease management for older adults with diabetes in primary care facilities in China, medication reviews for older patients with diabetes are limited to the antihyperglycemics they are currently taking (Li et al., 2017). Interventional studies aiming to optimize prescriptions for chronic polypharmacy and PIM use in older adults with diabetes in primary care facilities in China are also warranted.

## 6 Strengths and limitations

The strengths of our study are listed herein. A large-sample community-followed cohort of older adults with diabetes and SHDRDMCD covering medical records of 83 hospitals and 702 primary care centers in Shenzhen, China, were available. Three out of 5 categories of the 2019 Beers Criteria (including 39 out of 42 PIM items) were investigated. Only chronically used drugs were calculated for chronic polypharmacy assessment. The associated AHO of exposure to chronic polypharmacy and PIMs was explored simultaneously. The dose‒response relationship between AHO and chronic polypharmacy and PIMs was studied. To reduce the potential bias of the results of this study, the following limitations were unsettled. Categories IV and V of the 2019 Beers Criteria were not evaluated due to a lack of some laboratory data and information on the concurrent use of drugs. Older adults with diabetes who did not use any drugs or only used Chinese patent medicine were excluded from this study, which might lead to an overestimation of the prevalence of PIMs. Patients’ adherence could not be evaluated with an electronic medical record, which might overestimate or underestimate the risk of AHO in older adults with diabetes who are exposed to chronic polypharmacy and/or PIMs. The prevalence of chronic polypharmacy in this study was not comparable among studies with the definition of only the number of drugs or the fourth and fifth ATC levels. Finally, the incidence of hospital admission for heart failure and dementia was low during the 2-year follow-up, which might affect the power of the test.

In conclusion, chronic polypharmacy and PIMs were prevalent in older adults with diabetes in Chinese communities. Iatrogenic exposure to chronic polypharmacy and PIMs is associated with a higher incidence of different AHO. This observational evidence highlights the necessity of patient-centered medication reviews for chronic polypharmacy and PIM use in older patients with diabetes in primary care facilities in China and attracts attention for the caution of polypharmacy, especially PIM using in older adults with diabetes in clinical practice.

## Data Availability

The raw data supporting the conclusion of this article will be made available by the authors, without undue reservation.
