# Supplementary material for: Associated adverse health outcomes of polypharmacy and potentially inappropriate medications in community-dwelling older adults with diabetes
Source: Front Pharmacol. 2023 Nov 16;14:1284287. doi: 10.3389/fphar.2023.1284287 (PMC10687175; doi:10.3389/fphar.2023.1284287)
Supplement: Supplementary file 1 [file DataSheet1.docx]

# Appendices

Supplemental table 1. STROBE Statement—checklist of items that should be included in reports of observational studies.

|  | | **Item No** | **Recommendation** |
| --- | --- | --- | --- |
| **Title and abstract** | | 1 | (*a*) Indicate the study’s design with a commonly used term in the title or the abstract. **Yes, pages 1 and 3.** |
|  |  |  | (*b*) Provide in the abstract an informative and balanced summary of what was done and what was found. **Yes, pages 3-4.** |
| **Introduction** | | | |
| Background/rationale | | 2 | Explain the scientific background and rationale for the investigation being reported.  **Yes, pages 5-6.** |
| Objectives | | 3 | State specific objectives, including any prespecified hypotheses. **Yes, page 6.** |
| **Methods** | | | |
| Study design | | 4 | Present key elements of study design early in the paper. **Yes, page 7.** |
| Setting | | 5 | Describe the setting, locations, and relevant dates, including periods of recruitment, exposure, follow-up, and data collection. **Yes, pages 7 and 8.** |
| Participants | | 6 | (*a*) *Cohort study*—Give the eligibility criteria, and the sources and methods of selection of participants. Describe methods of follow-up. **Yes, pages 7 and 8.**  *Case-control study*—Give the eligibility criteria, and the sources and methods of case ascertainment and control selection. Give the rationale for the choice of cases and controls  *Cross-sectional study*—Give the eligibility criteria, and the sources and methods of selection of participants |
|  |  |  | (*b*) *Cohort study*—For matched studies, give matching criteria and number of exposed and unexposed  *Case-control study*—For matched studies, give matching criteria and the number of controls per case |
| Variables | | 7 | Clearly define all outcomes, exposures, predictors, potential confounders, and effect modifiers. Give diagnostic criteria, if applicable. **Yes, pages 8-10.** |
| Data sources/ measurement | | 8* | For each variable of interest, give sources of data and details of methods of assessment (measurement). Describe comparability of assessment methods if there is more than one group. **Yes, pages 7-10.** |
| Bias | | 9 | Describe any efforts to address potential sources of bias. **Yes, pages 7-11.** |
| Study size | | 10 | Explain how the study size was arrived at. **Yes, pages 7-8 and 11-12.** |
| Quantitative variables | | 11 | Explain how quantitative variables were handled in the analyses. If applicable, describe which groupings were chosen and why. **Yes, pages 7-11.** |
| Statistical methods | | 12 | (*a*) Describe all statistical methods, including those used to control for confounding. **Yes, pages 10 and 11.** |
|  |  |  | (*b*) Describe any methods used to examine subgroups and interactions. **Yes, pages 10 and 11.** |
|  |  |  | (*c*) Explain how missing data were addressed. **Yes, pages 10 and 11.** |
|  |  |  | (*d*) *Cohort study*—If applicable, explain how loss to follow-up was addressed.  **Yes, pages 10 and 11.**  *Case-control study*—If applicable, explain how matching of cases and controls was addressed  *Cross-sectional study*—If applicable, describe analytical methods taking account of sampling strategy. |
|  |  |  | (*e*) Describe any sensitivity analyses. **Yes, pages 10 and 11.** |
| **Results** | | | |
| Participants | 13* | (a) Report numbers of individuals at each stage of study—eg numbers potentially eligible, examined for eligibility, confirmed eligible, included in the study, completing follow-up, and analysed. **Yes, pages 11-12.** | |
|  |  | (b) Give reasons for non-participation at each stage. **Yes, page 11.** | |
|  |  | (c) Consider use of a flow diagram. **Yes, page 11.** | |
| Descriptive data | 14* | (a) Give characteristics of study participants (eg demographic, clinical, social) and information on exposures and potential confounders. **Yes, pages 11-12 and 22-24.** | |
|  |  | (b) Indicate number of participants with missing data for each variable of interest. **Yes, pages 11-12 and 22-24.** | |
|  |  | (c) *Cohort study*—Summarise follow-up time (eg, average and total amount) **No.** | |
| Outcome data | 15* | *Cohort study*—Report numbers of outcome events or summary measures over time. **Yes, pages 11-12.** | |
|  |  | *Case-control study—*Report numbers in each exposure category, or summary measures of exposure | |
|  |  | *Cross-sectional study—*Report numbers of outcome events or summary measures | |
| Main results | 16 | (*a*) Give unadjusted estimates and, if applicable, confounder-adjusted estimates and their precision (eg, 95% confidence interval). Make clear which confounders were adjusted for and why they were included. **Yes, pages 12-13.** | |
|  |  | (*b*) Report category boundaries when continuous variables were categorized. **Yes, pages 10, and 22-24.** | |
|  |  | (*c*) If relevant, consider translating estimates of relative risk into absolute risk for a meaningful time period. **No** | |
| Other analyses | 17 | Report other analyses done—eg analyses of subgroups and interactions, and sensitivity analyses. **Yes, pages 11-13.** | |
| **Discussion** | | | |
| Key results | 18 | Summarise key results with reference to study objectives. **Yes, pages 13-14.** | |
| Limitations | 19 | Discuss limitations of the study, taking into account sources of potential bias or imprecision. Discuss both direction and magnitude of any potential bias. **Yes, pages 13-16.** | |
| Interpretation | 20 | Give a cautious overall interpretation of results considering objectives, limitations, multiplicity of analyses, results from similar studies, and other relevant evidence. **Yes, pages 13-16.** | |
| Generalisability | 21 | Discuss the generalisability (external validity) of the study results. **Yes, pages 13-16.** | |
| **Other information** | | | |
| Funding | 22 | Give the source of funding and the role of the funders for the present study and, if applicable, for the original study on which the present article is based. **Yes, page 17.** | |

*Give information separately for cases and controls in case-control studies and, if applicable, for exposed and unexposed groups in cohort and cross-sectional studies.

**Note:** An Explanation and Elaboration article discusses each checklist item and gives methodological background and published examples of transparent reporting. The STROBE checklist is best used in conjunction with this article (freely available on the Web sites of PLoS Medicine at http://www.plosmedicine.org/, Annals of Internal Medicine at http://www.annals.org/, and Epidemiology at http://www.epidem.com/). Information on the STROBE Initiative is available at www.strobe-statement.org.

**Supplementary Table 2. The evaluated PIM exposure items and corresponding notes or reason for exclusion by using the 2019 American Geriatrics Society Beers criteria.**

|  | **Criteria** | **Drugs** | **Inclusion** | **Reason for exclusion/ Notes** |
| --- | --- | --- | --- | --- |
| **Category I: medications should be avoided by most older adults – Table 2. 2019 American Geriatrics Society Beers Criteria for Potentially Inappropriate Medication Use in Older Adults** | | | | |
| **Anticholigergics** | | | | |
| 1 | First-generation antihistamines | Brompheniramine  Carbinoxamine  Chlorpheniramine  Clemastine  Cyproheptadine  Dexbrompheniramine  Dexchlorpheniramine  Dimenhydrinate  Diphenhydramine (oral)  Doxylamine  Hydroxyzine  Meclizine  Promethazine  Pyrilamine  Triprolidine | Yes |  |
| 2 | Antiparkinsonian agents | Benztropine (oral)  Trihexyphenidyl | Yes | Excluded non-oral dosage forms of benztropine. |
| 3 | Antispasmodics | Atropine (excludes ophthalmic)  Belladonna alkaloids  Clidinium-chlordiazepoxide  Dicyclomine Homatropine  (excludes ophthalmic)  Hyoscyamine  Methscopolamine  Propantheline  Scopolamine | Yes | Excluded ophthalmic dosage forms of atropine and dicyclomine homatropine. Include combination medication contained belladonna alkaloids |
| 4 | Antithrombotics | Dipyridamole | Yes | Excluded extended-release combination with aspirin |
| 5 | Anti-infective | Nitrofurantoin | Yes | Not evaluated for the section that required lab data |
| **Cardiovascular** | | | | |
| 6 | Peripheral alpha-1 blockers | Doxazosin  Prazosin  Terazosin | Yes |  |
| 7 | Central alpha blockers | Clonidine  Guanabenz  Guanfacine  Methyldopa  Reserpine (> 0.1mg/day) | Yes | Excluded clonidine for first-line treatment of hypertension/excluded reserpine, which required data for dosage by day |
| 8 |  | Disopyramide | Yes |  |
| 9 |  | Dronedarone | Yes | Avoid for atrial fibrillation (ICD-10: I48) or heart failure (ICD-10: I50) |
| 10 |  | Digoxin | Yes |  |
| 11 |  | Nifedipine | Yes | Excluded sustained and controlled release dosage form of nifedipine |
| 12 |  | Amiodarone | Yes | Avoid for atrial fibrillation (ICD-10: I48) except heart failure (ICD-10: I50) or substantial left ventricular hypertrophy (ICD-10: I51.701) |
| **Central nervous systems** | | | | |
| 13 | Antidepressants | Amitriptyline | Yes |  |
|  |  | Amoxapine | Yes |  |
|  |  | Clomipramine | Yes |  |
|  |  | Desipramine | Yes |  |
|  |  | Doxepin > 6 mg/day | No | Excluded doxepin for lacking dosage data |
|  |  | Imipramine | Yes |  |
|  |  | Nortriptyline | Yes |  |
|  |  | Paroxetine | Yes |  |
|  |  | Protriptyline | Yes |  |
|  |  | Trimipramine | Yes |  |
| 14 | Antipsychotics | First generation (conventional)  Perphenazine  Chlorpromazine  Penfluridol  Sulpiride  Tiapride  Haloperidol  Amisulpride  Second generation (atypical)  Aripiprazole  Clozapine  Olanzapine  Paliperidone  Quetiapine  Risperidone  Ziprasidone | Yes | Any diagnoses of schizophrenia (ICD-10: F20, F21, F23.2, F25) and bipolar disorders (ICD-10: F30, F31) during the study period were considered appropriate use. Appropriate use for drugs used as antiemetic during chemotherapy were not evaluated (chlorpromazine and perphenazine) |
| 15 | Barbiturates | Amobarbital  Butabarbital  Butalbital  Mephobarbital  Pentobarbital  Phenobarbital  Secobarbital | Yes |  |
| 16 | Benzodiazepines | Alprazolam  Estazolam  Lorazepam  Oxazepam  Temazepam  Triazolam  Chlordiazepoxide (alone or in  combination with amitriptyline or  clidinium)  Clonazepam  Clorazepate  Diazepam  Flurazepam  Quazepam | Yes |  |
| 17 |  | Meprobamate | Yes |  |
| 18 | Nonbenzodiazepine, benzodiazepine receptor agonist hypnotics | Eszopiclone  Zolpidem  Zaleplon | Yes |  |
| 19 |  | Ergoloid mesylates  Isoxsuprine | Yes |  |
| **Endocrine** | | | | |
| 20 | Androgens | Methyltestosterone  Testosterone | Yes |  |
| 21 |  | Desiccated thyroid | Yes |  |
| 22 |  | Estrogens with or without progestins | Yes | Excluded vaginal form/Included combinations contained estrogen |
| 23 | Growth hormone |  | Yes |  |
| 24 | Insulin, sliding scale |  | Yes | Included insulin regimens containing only short- or rapid-acting insulin dosed according to current blood glucose levels in first prescription without concurrent use of basal or long-acting insulin |
| 25 |  | Megestrol | Yes |  |
| 26 | Sulfonylureas | Chlorpropamide  Glimepiride | Yes |  |
|  |  | Glyburide |  |  |
| **Gastrointestinal** | | | | |
| 27 |  | Metoclopramide | Yes | Any diagnoses of gastroparesis (ICD-10: K31.809, K31.819, K31.901) in the baseline and assessment periods were considered appropriate use |
| 28 |  | Mineral oil, given orally | Yes |  |
| 29 | Proton-pump inhibitors | [Omeprazole](https://db.yaozh.com/atc?atc_num=A02BC01)  [Rabeprazole](https://db.yaozh.com/atc?atc_num=A02BC04)  [Pantoprazole](https://db.yaozh.com/atc?atc_num=A02BC02)  [Lansoprazole](https://db.yaozh.com/atc?atc_num=A02BC03)  [Esomeprazole](https://db.yaozh.com/atc?atc_num=A02BC05)  Ilaprazole | Yes | Included scheduled use for > 8 weeks |
| **Pain medications** | | | | |
| 30 |  | Meperidine | Yes |  |
| 31 | NSAIDs | Aspirin >325 mg/d (exclude) | No | Excluded because dosage was required |
|  |  | Diclofenac  Diflunisal  Etodolac  Fenoprofen  Ibuprofen  Ketoprofen  Meclofenamate  Mefenamic acid  Meloxicam  Nabumetone  Naproxen  Oxaprozin  Piroxicam  Sulindac  Tolmetin | Yes |  |
| 32 |  | Indomethacin  Ketorolac | Yes  Yes |  |
| 33 | Skeletal muscle relaxants | Carisoprodol  Chlorzoxazone  Cyclobenzaprine  Metaxalone  Methocarbamol  Orphenadrine | Yes |  |
| **Genitourinary** | | | | |
| 34 |  | Desmopressin | Yes | Any diagnoses of nocturia or nocturnal polyuria (ICD-10: R35.x00) in the baseline and assessment periods were considered PIM |
| **Category II: medications should be avoided by older adults with certain conditions – Table 3. 2019 American Geriatrics Society Beers Criteria for Potentially Inappropriate Medication Use in Older Adults Due to Drug-Disease or Drug-Syndrome Interaction That May Exacerbate the Disease or Syndrome** | | | | |
|  | Disease or syndrome | Drugs |  |  |
| 35 | Heart failure (ICD-10: I50) | Cilostazol | Yes |  |
|  |  | Nondihydropyridine CCBs (avoid only for heart failure with reduced ejection fraction) | No | Excluded because specific clinical features were required |
|  |  | NSAIDS and COX-2 inhibitors | Yes |  |
|  |  | Thiazolidinediones (pioglitazone, rosiglitazone) | Yes |  |
|  |  | Dronedarone | Yes |  |
| 36 | Dementia or cognitive impairment (ICD-10: F00.0-F01.9, F02, F03) | Anticholinergics: observe in category I “Anticholinergics”  Benzodiazepine: observe in category I “Benzodiazepine”  Nonbenzodiazepine, Benzodiazepine receptor agonist Hypnotics: observe in category I “Nonbenzodiazepine, Benzodiazepine receptor agonist Hypnotics”  Antipsychotics: observe in category I “Antipsychotics” | Yes |  |
| 37 | History of falls or fractures* (ICD-10: D48.9, M80, M84, S02, S12, S22, S32, S42, S52, S62, S72, S82, S92, T90, T91, T92, T93, T94, W00-W19) | Antiepileptics:  Midazolam  Diazepam  Phenobarbital  Chloral hydrate  Magnesium sulphate  Antipsychotics: observe in category I “Antipsychotics”  Benzodiazepine: observe in category I “Benzodiazepine”  Nonbenzodiazepine, Benzodiazepine receptor agonist Hypnotics: observe in category I “Nonbenzodiazepine, Benzodiazepine receptor agonist Hypnotics”  TCAs: observe in category III  SSRIs: observe in category III  SNRIs: observe in category III  Opioids | Yes | Excludes opioids for pain management due to recent fractures or joint replacement |
| 38 | Parkinson disease (ICD-10: G20-G21) | Antiemetics  Metoclopramide  Prochlorperazine  Promethazine  All antipsychotics (except quetiapine, clozapine, pimavanserin) | Yes |  |
| 39 | History of gastric or duodenal ulcers (ICD-10: K25-K27) | Aspirin (>325 mg/d) (exclude) | No | Excluded because dosage was required |
|  |  | Non-COX-2 selective NSAIDs | No | Excluded because we could not ensure that the patient concurrently used PPI or misoprostol |
| 40 | Chronic kidney disease stage IV or higher (creatinine clearance <30 mL/min) | NSAIDs | No | Excluded because laboratory data and disease severity were required |
| 41 | Urinary incontinence (all types) in women (ICD-10: R32, N39) | Estrogen oral and transdermal (excludes intravaginal estrogen)  Peripheral alpha-1 blockers  Doxazonsin  Prazosin  Terazosin | Yes | Sex limited to female and excluded intravaginal estrogen |
| 42 | Lower urinary tract symptoms, benign prostatic hyperplasia for male (ICD-10: R30.901, R35.x51, F45.354, R32, N39, R30.001, N40) | Strongly anticholinergic drugs, except antimuscarinics for urinary incontinence  Darifenacin  Fesoterodine  Flavoxate  Oxybutynin  Solifenacin  Tolterodine  Trospium | Yes | Sex limited to male |
| **Category III: medications that should be used with caution - Table 4. 2019 American Geriatrics Society Beers Criteria for Potentially Inappropriate Medications to Be Used with Caution in Older Adults** | | | | |
| 43 | Aspirin for primary prevention of cardiovascular disease and colorectal cancer |  | No | Excluded because detailed clinical information was required |
| 44 | Dabigatran |  | Yes | Patients aged ≥75 |
|  | Rivaroxaban |  | Yes | Patients aged ≥75 |
| 45 | Prasugrel |  | Yes | Patients aged ≥75 |
| 46 | Antipsychotics | observe in category I “Antipsychotics” | Yes |  |
| 47 | Carbamazepine |  | Yes |  |
| 48 | Diuretics | Spironolactone  Indapamide  Torasemide  Furosemide  Hydrochlorothiazide  Amiloride  Bumetanide  Tolvaptan | Yes |  |
| 49 | Mirtazapine |  | Yes |  |
| 50 | Oxcarbazepine |  | Yes |  |
| 51 | SNRIs | Venlafaxine  Duloxetine | Yes |  |
| 52 | SSRIs | Citalopram  Fluvoxamine  Fluoxetine  Paroxetine  Sertraline | Yes |  |
| 53 | TCAs | Amitriptyline  Doxepin  Clomipramine | Yes |  |
| 54 | Tramadol |  | Yes |  |
| 55 | Dextromethorphan/quinidine |  | Yes |  |
| 56 | Trimethoprim- sulfamethoxazole |  | No | Excluded because of lacking clinical information and lab data were required |

Abbreviations: PIMs = potentially inappropriate medications, NSAIDs = non-steroidal anti-inflammatory drugs, COX-2 = cyclooxygenase-2, SNRI = serotonin-norepinephrine reuptake inhibitor, SSRI = selective serotonin reuptake inhibitor, TCA = tricyclic antidepressant.

**Supplemental table 3. The International Classification of Disease, 10^th^ revision (ICD-10) codes for the comorbidities included for medication intensity analysis.**

| **NO.** | **Disease** | **ICD-10 codes** |
| --- | --- | --- |
| 1 | cardiovascular disease | I05.0-I08.9, I20.0-I22.9, I24.0, I24.8-I25.2, I25.5-I25.9, I34.0-I37.9, I47, I48, I49.8, I49.9, I50.0-I50.9, I70.9 |
| 2 | cerebrovascular disease | I60.0-I61.9, I63.0-I63.9, I64, I65.0-I66.9, I67 |
| 3 | hypertension | I10, I11, I12, I13, I15, I15.0, I15.1, I15.2, I15.8, I15.9 |
| 4 | hyperlipidemia | E78.0-E78.5 |
| 5 | tumor | C00.0-C13.9, C15.0-C25.9, C30.0-C34.9, C37.0-C38.8, C40.0-C41.9, C43.0-C45.9, C47.0-C54.9, C56.0-C57.8, C58, C60.0-C63.8, C64.0-C67.9, C68.0-C68.8, C69.0-C75.8, C81.0-C86.6, C88.0-C97.9, D00.0-D00.2, D01.0-D01.3, D02.0-D02.3, D03.0-D06.9, D07.0-D07.2, D07.4, D07.5, D09.0, D09.2-D09.8, D10.0-D10.7, D11.0-D12.9, D13.0-D13.7, D14.0-D14.32, D15.0-D25.9, D26.0, D26.1, D27.0-D27.9, D28.0- D28.7, D29.0-D29.8, D30.0-D30.8, D31.0-D36.7, D37.0-D37.5, D38.0-D38.5, D39.1-D39.8, D40.0-D40.8, D41.0-D41.8, D42.0-D43.9, D44.0-D44.8, D45.0-D47.9, D48.0-D48.7, D49.2-D49.4, D49.6, D49.8, K31.7, K62.0, K62.1, K63.5, N60.0-N60.9, N84.0-N84.8, N87.0-N87.9, Z03.1, Z08.0-Z09.9, Z12.0-Z12.9, Z80.0-Z80.9, Z85.0-Z85.9, Z86.0 |
| 6 | chronic liver disease | B18.0-B18.8, K70.0-K70.9, K72.1, K73.0-K73.9, K74.0-K74.6, K76.0, K77 |
| 7 | chronic pulmonary disease | A15.3, J18.0-J18.9, J41.0, J41.1, J41.8, J44.0-J44.9, J45.9, J47.0-J47.9, |
| 8 | chronic kidney disease | E10.2, E11.2, E12.2, E13.2, E14.2, I12.0-I13.9, N02.0-N08.8, N15.0, N18.0-N18.9, Z49.0-Z49.3, Z52.4, Z99.2 |
| 9 | chronic gastrointestinal disease | K25.4-K25.7, K26.4-K26.7, K27.4-K27.7, K28.4-K28.7, K29.5, K50.0-K51.3, K81.1, K86.0, K86.1, |
| 10 | osteoarthritis and rheumatoid arthritis | M13.0-M13.9, M15.0-M19.9; M05.0-M06.9, M08.0-M08.8 |

Abbreviations: ICD-10, International Statistical Classification of Diseases, 10^th^ Revision.

**Supplemental table 4. Sensitivity analysis before and after multiple interpolation of multivariate adjusted logistics regression of associated adverse health outcomes of polypharmacy for community-followed older adults with diabetes.**

|  | **Multivariate adjusted logistic regression**  **(multiple interpolation)** | | |  | **Multivariate adjusted logistic regression (2695 samples missing)** | | |
| --- | --- | --- | --- | --- | --- | --- | --- |
|  | **OR** | **95%CI** | ***P*** |  | **aOR** | **95%CI** | ***P*** |
| **All-cause hospital admission** |  |  |  |  |  |  |  |
| moderate | 1.95 | (1.76, 2.17) | < 0.001 |  | 1.92 | (1.68, 2.20) | < 0.001 |
| severe | 2.86 | (2.38, 3.43) | < 0.001 |  | 2.93 | (2.33, 3.70) | < 0.001 |
| **Hospital admission for coronary heart disease** |  |  |  |  |  |  |  |
| moderate | 2.00 | (1.59, 2.53) | < 0.001 |  | 1.83 | (1.36, 2.47) | < 0.001 |
| severe | 5.76 | (4.28, 7.78) | < 0.001 |  | 4.86 | (3.31, 7.17) | < 0.001 |
| **Hospital admission for stroke** |  |  |  |  |  |  |  |
| moderate | 2.05 | (1.62, 2.60) | < 0.001 |  | 1.96 | (1.43, 2.70) | < 0.001 |
| severe | 2.48 | (1.78, 3.47) | < 0.001 |  | 2.28 | (1.45, 3.57) | < 0.001 |
| **Hospital admission for dementia** |  |  |  |  |  |  |  |
| moderate | 1.80 | (1.08, 3.05) | 0.025 |  | 1.74 | (1.00, 3.18) | 0.047 |
| severe | 3.61 | (1.84, 7.15) | < 0.001 |  | 3.67 | (1.63, 8.32) | 0.001 |
| **Hospital admission for heart failure** |  |  |  |  |  |  |  |
| moderate | 0.69 | (0.30, 1.58) | 0.369 |  | 0.81 | (0.43, 1.68) | 0.238 |
| severe | 1.59 | (0.62, 4.25) | 0.343 |  | 1.94 | (0.71, 4.73) | 0.523 |
| **Emergency department admission** |  |  |  |  |  |  |  |
| moderate | 1.38 | (1.23, 1.55) | < 0.001 |  | 1.35 | (1.16, 1.56) | < 0.001 |
| severe | 1.75 | (1.45, 2.10) | < 0.001 |  | 1.73 | (1.36, 2.19) | < 0.001 |
| **Bone fracture** |  |  |  |  |  |  |  |
| moderate | 0.93 | (0.74, 1.17) | 0.539 |  | 0.79 | (0.59, 1.07) | 0.128 |
| severe | 1.13 | (0.80, 1.59) | 0.484 |  | 0.87 | (0.56,1.37) | 0.531 |
| **Constipation** |  |  |  |  |  |  |  |
| moderate | 1.19 | (0.97, 1.45) | 0.119 |  | 1.18 | (0.93, 1.50) | 0.173 |
| severe | 1.22 | (0.92, 1.61) | 0.166 |  | 1.21 | (0.84, 1.73) | 0.295 |
| **Diarrhea** |  |  |  |  |  |  |  |
| moderate | 0.80 | (0.52, 1.24) | 0.320 |  | 0.66 | (0.38, 1.14) | 0.133 |
| severe | 0.91 | (0.49, 1.67) | 0.765 |  | 0.86 | (0.41, 1.79) | 0.662 |

Abbreviations: OR = odds ratio, aOR = adjusted odds ratio, 95%CI = 95% confidence interval.

**Supplemental table 5. Sensitivity analysis before and after multiple interpolation of multivariate adjusted logistics regression of associated adverse health outcomes of potentially inappropriate medication for community-followed older adults with diabetes.**

|  | **Multivariate adjusted logistic regression**  **(multiple interpolation)** | | |  | **Multivariate adjusted logistic regression (2695 samples missing)** | | |
| --- | --- | --- | --- | --- | --- | --- | --- |
|  | **OR** | **95%CI** | ***P*** |  | **aOR** | **95%CI** | ***P*** |
| **All-cause hospital admission** |  |  |  |  |  |  |  |
| 1 | 1.22 | (1.10, 1.35) | < 0.001 |  | 1.24 | (1.09, 1.42) | 0.001 |
| 2 | 1.38 | (1.21, 1.57) | < 0.001 |  | 1.41 | (1.20, 1.67) | < 0.001 |
| ≥3 | 1.62 | (1.39, 1.87) | < 0.001 |  | 1.66 | (1.37, 2.01) | < 0.001 |
| **Hospital admission for coronary heart disease** |  |  |  |  |  |  |  |
| 1 | 1.28 | (0.96, 1.66) | 0.123 |  | 1.34 | (1.01, 1.78) | 0.407 |
| 2 | 1.11 | (0.86, 1.43) | 0.411 |  | 1.20 | (0.86, 1.66) | 0.287 |
| ≥3 | 1.04 | (0.80, 1.36) | 0.758 |  | 1.15 | (0.82, 1.62) | 0.495 |
| **Hospital admission for stroke** |  |  |  |  |  |  |  |
| 1 | 1.06 | (0.85, 1.33) | 0.605 |  | 1.13 | (0.83, 1.55) | 0.436 |
| 2 | 1.16 | (0.89, 1.51) | 0.266 |  | 1.29 | (0.91, 1.83) | 0.148 |
| ≥3 | 1.35 | (1.03, 1.77) | 0.033 |  | 1.58 | (1.09, 2.30) | 0.016 |
| **Hospital admission for dementia** |  |  |  |  |  |  |  |
| 1 | 1.69 | (0.93, 2.91) | 0.141 |  | 1.49 | (0.81, 2.76) | 0.203 |
| 2 | 1.66 | (0.87, 3.01) | 0.182 |  | 1.47 | (0.73, 2.96) | 0.275 |
| ≥3 | 1.40 | (0.76, 2.62) | 0.283 |  | 1.43 | (0.67, 3.03) | 0.355 |
| **Hospital admission for heart failure** |  |  |  |  |  |  |  |
| 1 | 2.09 | (0.80, 5.84) | 0.139 |  | 1.97 | (0.72, 5.82) | 0.239 |
| 2 | 3.17 | (1.13, 9.37) | 0.03 |  | 3.04 | (1.06, 9.76) | 0.041 |
| ≥3 | 6.97 | (2.60, 20.48) | < 0.001 |  | 6.76 | (2.37, 21.83) | < 0.001 |
| **Emergency department admission** |  |  |  |  |  |  |  |
| 1 | 1.66 | (1.49, 1.86) | < 0.001 |  | 1.63 | (1.41, 1.90) | < 0.001 |
| 2 | 1.99 | (1.73, 2.28) | < 0.001 |  | 1.99 | (1.67, 2.39) | < 0.001 |
| ≥3 | 2.92 | (2.50, 3.40) | < 0.001 |  | 2.70 | (2.21, 3.29) | < 0.001 |
| **Bone fracture** |  |  |  |  |  |  |  |
| 1 | 1.82 | (1.44, 2.31) | < 0.001 |  | 1.48 | (1.10, 2.00) | 0.01 |
| 2 | 2.13 | (1.61, 2.81) | < 0.001 |  | 1.66 | (1.16, 2.39) | 0.005 |
| ≥3 | 2.73 | (2.03, 3.67) | < 0.001 |  | 2.68 | (1.85, 3.89) | < 0.001 |
| **Constipation** |  |  |  |  |  |  |  |
| 1 | 1.18 | (0.97, 1.43) | 0.125 |  | 1.12 | (0.88, 1.43) | 0.346 |
| 2 | 1.43 | (1.14, 1.78) | 0.002 |  | 1.35 | (1.02, 1.78) | 0.035 |
| ≥3 | 2.00 | (1.59, 2.52) | < 0.001 |  | 1.98 | (1.47, 2.65) | < 0.001 |
| **Diarrhea** |  |  |  |  |  |  |  |
| 1 | 1.12 | (0.69, 1.80) | 0.656 |  | 1.27 | (0.69, 2.35) | 0.436 |
| 2 | 2.70 | (1.68, 4.34) | < 0.001 |  | 3.28 | (1.82, 5.99) | < 0.001 |
| ≥3 | 2.78 | (1.64, 4.73) | < 0.001 |  | 3.44 | (1.78, 6.73) | < 0.001 |

Abbreviations: OR = odds ratio, aOR = adjusted odds ratio, 95%CI = 95% confidence interval.

**Supplemental table 6. Generalized variance-inflation factors of adjusted logistic regression models for all-cause hospital admission.**

| **Variables** | **All-cause hospital admission** | | |
| --- | --- | --- | --- |
|  | **GVIF** | **df** | **GVIF^(1/(2*df))** |
| Age | 1.052 | 3 | 1.008 |
| Gender | 1.028 | 1 | 1.014 |
| Potentially inappropriate medication | 1.464 | 3 | 1.065 |
| Polypharmacy | 2.009 | 2 | 1.191 |
| Systolic blood pressure | 1.275 | 1 | 1.129 |
| Diastolic blood pressure | 1.224 | 1 | 1.106 |
| Body mass index | 1.014 | 1 | 1.007 |
| Fasting plasma glucose | 1.479 | 1 | 1.216 |
| 2-hour postprandial blood glucose | 1.450 | 1 | 1.204 |
| Glycated hemoglobin | 1.137 | 1 | 1.066 |
| Diabetic peripheral disease | 1.049 | 1 | 1.024 |
| Diabetic kidney disease | 1.128 | 1 | 1.062 |
| Diabetic retinal | 1.042 | 1 | 1.021 |
| Chronic respiratory disease | 1.044 | 1 | 1.022 |
| Osteoarthritis and rheumatoid arthritis | 1.076 | 1 | 1.037 |
| Chronic liver disease | 1.016 | 1 | 1.008 |
| Hypertension | 1.262 | 1 | 1.123 |
| Hyperlipemia | 1.238 | 1 | 1.113 |
| Cerebrovascular disease | 1.058 | 1 | 1.028 |
| Chronic kidney disease | 1.138 | 1 | 1.067 |
| Chronic gastrointestinal disease | 1.018 | 1 | 1.009 |
| Cardiovascular disease | 1.257 | 1 | 1.121 |
| Tumor | 1.010 | 1 | 1.005 |

Abbreviations: GVIF = generalized variance-inflation factor, df = degree of freedom.

**Supplemental table 7. Generalized variance-inflation factors of adjusted logistic regression models for hospital admission for coronary heart disease.**

| **Variables** | **Hospital admission for coronary heart disease** | | |
| --- | --- | --- | --- |
|  | **GVIF** | **df** | **GVIF^(1/(2*df))** |
| Age | 1.104 | 3 | 1.016 |
| Gender | 1.035 | 1 | 1.017 |
| Potentially inappropriate medication | 1.682 | 3 | 1.091 |
| Polypharmacy | 2.224 | 2 | 1.221 |
| Systolic blood pressure | 1.248 | 1 | 1.117 |
| Diastolic blood pressure | 1.201 | 1 | 1.096 |
| Body mass index | 1.018 | 1 | 1.009 |
| Fasting plasma glucose | 1.552 | 1 | 1.246 |
| 2-hour postprandial blood glucose | 1.549 | 1 | 1.244 |
| Glycated hemoglobin | 1.177 | 1 | 1.085 |
| Diabetic peripheral disease | 1.156 | 1 | 1.075 |
| Diabetic kidney disease | 1.359 | 1 | 1.166 |
| Diabetic retinal | 1.132 | 1 | 1.064 |
| Chronic respiratory disease | 1.091 | 1 | 1.044 |
| Osteoarthritis and rheumatoid arthritis | 1.106 | 1 | 1.052 |
| Chronic liver disease | 1.033 | 1 | 1.017 |
| Hypertension | 1.254 | 1 | 1.120 |
| Hyperlipemia | 1.213 | 1 | 1.102 |
| Cerebrovascular disease | 1.095 | 1 | 1.047 |
| Chronic kidney disease | 1.317 | 1 | 1.148 |
| Chronic gastrointestinal disease | 1.034 | 1 | 1.017 |
| Cardiovascular disease | 1.220 | 1 | 1.104 |
| Tumor | 1.020 | 1 | 1.010 |

Abbreviations: GVIF = generalized variance-inflation factor, df = degree of freedom.

**Supplemental table 8. Generalized variance-inflation factors of adjusted logistic regression models for hospital admission for stroke.**

| **Variables** | **Hospital admission for stroke** | | |
| --- | --- | --- | --- |
|  | **GVIF** | **df** | **GVIF^(1/(2*df))** |
| Age | 1.098 | 3 | 1.016 |
| Gender | 1.033 | 1 | 1.017 |
| Potentially inappropriate medication | 1.650 | 3 | 1.087 |
| Polypharmacy | 2.232 | 2 | 1.222 |
| Systolic blood pressure | 1.286 | 1 | 1.134 |
| Diastolic blood pressure | 1.247 | 1 | 1.117 |
| Body mass index | 1.019 | 1 | 1.010 |
| Fasting plasma glucose | 1.479 | 1 | 1.216 |
| 2-hour postprandial blood glucose | 1.463 | 1 | 1.210 |
| Glycated hemoglobin | 1.157 | 1 | 1.076 |
| Diabetic peripheral disease | 1.158 | 1 | 1.076 |
| Diabetic kidney disease | 1.365 | 1 | 1.168 |
| Diabetic retinal | 1.154 | 1 | 1.074 |
| Chronic respiratory disease | 1.096 | 1 | 1.047 |
| Osteoarthritis and rheumatoid arthritis | 1.120 | 1 | 1.058 |
| Chronic liver disease | 1.042 | 1 | 1.021 |
| Hypertension | 1.216 | 1 | 1.103 |
| Hyperlipemia | 1.228 | 1 | 1.108 |
| Cerebrovascular disease | 1.122 | 1 | 1.060 |
| Chronic kidney disease | 1.321 | 1 | 1.149 |
| Chronic gastrointestinal disease | 1.026 | 1 | 1.013 |
| Cardiovascular disease | 1.289 | 1 | 1.135 |
| Tumor | 1.026 | 1 | 1.013 |

Abbreviations: GVIF = generalized variance-inflation factor, df = degree of freedom.

**Supplemental table 9. Generalized variance-inflation factors of adjusted logistic regression models for hospital admission for dementia.**

| **Variables** | **Hospital admission for dementia** | | |
| --- | --- | --- | --- |
|  | **GVIF** | **df** | **GVIF^(1/(2*df))** |
| Age | 1.080 | 3 | 1.013 |
| Gender | 1.049 | 1 | 1.024 |
| Potentially inappropriate medication | 1.872 | 3 | 1.110 |
| Polypharmacy | 2.608 | 2 | 1.271 |
| Systolic blood pressure | 1.243 | 1 | 1.115 |
| Diastolic blood pressure | 1.216 | 1 | 1.103 |
| Body mass index | 1.028 | 1 | 1.014 |
| Fasting plasma glucose | 1.574 | 1 | 1.254 |
| 2-hour postprandial blood glucose | 1.564 | 1 | 1.251 |
| Glycated hemoglobin | 1.147 | 1 | 1.071 |
| Diabetic peripheral disease | 1.133 | 1 | 1.064 |
| Diabetic kidney disease | 1.299 | 1 | 1.140 |
| Diabetic retinal | 1.068 | 1 | 1.034 |
| Chronic respiratory disease | 1.147 | 1 | 1.071 |
| Osteoarthritis and rheumatoid arthritis | 1.163 | 1 | 1.078 |
| Chronic liver disease | 1.074 | 1 | 1.036 |
| Hypertension | 1.335 | 1 | 1.155 |
| Hyperlipemia | 1.305 | 1 | 1.142 |
| Cerebrovascular disease | 1.182 | 1 | 1.087 |
| Chronic kidney disease | 1.340 | 1 | 1.158 |
| Chronic gastrointestinal disease | 1.054 | 1 | 1.027 |
| Cardiovascular disease | 1.450 | 1 | 1.204 |
| Tumor | 1.038 | 1 | 1.019 |

Abbreviations: GVIF = generalized variance-inflation factor, df = degree of freedom.

**Supplemental table 10. Generalized variance-inflation factors of adjusted logistic regression models for hospital admission for heart failure.**

| **Variables** | **Hospital admission for heart failure** | | |
| --- | --- | --- | --- |
|  | **GVIF** | **df** | **GVIF^(1/(2*df))** |
| Age | 1.247 | 3 | 1.037 |
| Gender | 1.086 | 1 | 1.042 |
| Potentially inappropriate medication | 1.889 | 3 | 1.112 |
| Polypharmacy | 2.364 | 2 | 1.239 |
| Systolic blood pressure | 1.411 | 1 | 1.187 |
| Diastolic blood pressure | 1.274 | 1 | 1.128 |
| Body mass index | 1.037 | 1 | 1.018 |
| Fasting plasma glucose | 1.478 | 1 | 1.216 |
| 2-hour postprandial blood glucose | 1.376 | 1 | 1.173 |
| Glycated hemoglobin | 1.171 | 1 | 1.082 |
| Diabetic peripheral disease | 1.000 | 1 | 1.000 |
| Diabetic kidney disease | 1.465 | 1 | 1.210 |
| Diabetic retinal | 1.093 | 1 | 1.045 |
| Chronic respiratory disease | 1.122 | 1 | 1.059 |
| Osteoarthritis and rheumatoid arthritis | 1.167 | 1 | 1.080 |
| Chronic liver disease | 1.052 | 1 | 1.026 |
| Hypertension | 1.187 | 1 | 1.089 |
| Hyperlipemia | 1.233 | 1 | 1.110 |
| Cerebrovascular disease | 1.145 | 1 | 1.070 |
| Chronic kidney disease | 1.519 | 1 | 1.232 |
| Chronic gastrointestinal disease | 1.102 | 1 | 1.050 |
| Cardiovascular disease | 1.259 | 1 | 1.122 |
| Tumor | 1.026 | 1 | 1.013 |

Abbreviations: GVIF = generalized variance-inflation factor, df = degree of freedom.

**Supplemental table 11. Generalized variance-inflation factors of adjusted logistic regression models for emergency department admission.**

| **Variables** | **Emergency department admission** | | |
| --- | --- | --- | --- |
|  | **GVIF** | **df** | **GVIF^(1/(2*df))** |
| Age | 1.053 | 3 | 1.009 |
| Gender | 1.036 | 1 | 1.018 |
| Potentially inappropriate medication | 1.471 | 3 | 1.067 |
| Polypharmacy | 2.039 | 2 | 1.195 |
| Systolic blood pressure | 1.250 | 1 | 1.118 |
| Diastolic blood pressure | 1.198 | 1 | 1.095 |
| Body mass index | 1.015 | 1 | 1.007 |
| Fasting plasma glucose | 1.476 | 1 | 1.215 |
| 2-hour postprandial blood glucose | 1.461 | 1 | 1.209 |
| Glycated hemoglobin | 1.141 | 1 | 1.068 |
| Diabetic peripheral disease | 1.105 | 1 | 1.051 |
| Diabetic kidney disease | 1.223 | 1 | 1.106 |
| Diabetic retinal | 1.095 | 1 | 1.047 |
| Chronic respiratory disease | 1.042 | 1 | 1.021 |
| Osteoarthritis and rheumatoid arthritis | 1.071 | 1 | 1.035 |
| Chronic liver disease | 1.021 | 1 | 1.010 |
| Hypertension | 1.252 | 1 | 1.119 |
| Hyperlipemia | 1.229 | 1 | 1.109 |
| Cerebrovascular disease | 1.058 | 1 | 1.028 |
| Chronic kidney disease | 1.183 | 1 | 1.087 |
| Chronic gastrointestinal disease | 1.022 | 1 | 1.011 |
| Cardiovascular disease | 1.234 | 1 | 1.111 |
| Tumor | 1.015 | 1 | 1.007 |

Abbreviations: GVIF = generalized variance-inflation factor, df = degree of freedom.

**Supplemental table 12. Generalized variance-inflation factors of adjusted logistic regression models for bone fracture.**

| **Variables** | **Bone fracture** | | |
| --- | --- | --- | --- |
|  | **GVIF** | **df** | **GVIF^(1/(2*df))** |
| Age | 1.090 | 3 | 1.015 |
| Gender | 1.039 | 1 | 1.019 |
| Potentially inappropriate medication | 1.721 | 3 | 1.095 |
| Polypharmacy | 2.477 | 2 | 1.254 |
| Systolic blood pressure | 1.265 | 1 | 1.125 |
| Diastolic blood pressure | 1.215 | 1 | 1.102 |
| Body mass index | 1.017 | 1 | 1.009 |
| Fasting plasma glucose | 1.546 | 1 | 1.244 |
| 2-hour postprandial blood glucose | 1.537 | 1 | 1.239 |
| Glycated hemoglobin | 1.159 | 1 | 1.077 |
| Diabetic peripheral disease | 1.174 | 1 | 1.083 |
| Diabetic kidney disease | 1.397 | 1 | 1.182 |
| Diabetic retinal | 1.135 | 1 | 1.065 |
| Chronic respiratory disease | 1.118 | 1 | 1.057 |
| Osteoarthritis and rheumatoid arthritis | 1.114 | 1 | 1.055 |
| Chronic liver disease | 1.035 | 1 | 1.017 |
| Hypertension | 1.277 | 1 | 1.130 |
| Hyperlipemia | 1.279 | 1 | 1.131 |
| Cerebrovascular disease | 1.126 | 1 | 1.061 |
| Chronic kidney disease | 1.382 | 1 | 1.175 |
| Chronic gastrointestinal disease | 1.027 | 1 | 1.014 |
| Cardiovascular disease | 1.336 | 1 | 1.155 |
| Tumor | 1.028 | 1 | 1.013 |

Abbreviations: GVIF = generalized variance-inflation factor, df = degree of freedom.

**Supplemental table 13. Generalized variance-inflation factors of adjusted logistic regression models for constipation.**

| **Variables** | **Constipation** | | |
| --- | --- | --- | --- |
|  | **GVIF** | **df** | **GVIF^(1/(2*df))** |
| Age | 1.073 | 3 | 1.012 |
| Gender | 1.032 | 1 | 1.016 |
| Potentially inappropriate medication | 1.667 | 3 | 1.089 |
| Polypharmacy | 2.377 | 2 | 1.241 |
| Systolic blood pressure | 1.255 | 1 | 1.120 |
| Diastolic blood pressure | 1.196 | 1 | 1.093 |
| Body mass index | 1.013 | 1 | 1.006 |
| Fasting plasma glucose | 1.426 | 1 | 1.194 |
| 2-hour postprandial blood glucose | 1.406 | 1 | 1.186 |
| Glycated hemoglobin | 1.125 | 1 | 1.061 |
| Diabetic peripheral disease | 1.109 | 1 | 1.053 |
| Diabetic kidney disease | 1.222 | 1 | 1.106 |
| Diabetic retinal | 1.078 | 1 | 1.038 |
| Chronic respiratory disease | 1.096 | 1 | 1.046 |
| Osteoarthritis and rheumatoid arthritis | 1.103 | 1 | 1.051 |
| Chronic liver disease | 1.034 | 1 | 1.017 |
| Hypertension | 1.249 | 1 | 1.117 |
| Hyperlipemia | 1.268 | 1 | 1.126 |
| Cerebrovascular disease | 1.105 | 1 | 1.051 |
| Chronic kidney disease | 1.230 | 1 | 1.109 |
| Chronic gastrointestinal disease | 1.034 | 1 | 1.017 |
| Cardiovascular disease | 1.304 | 1 | 1.142 |
| Tumor | 1.021 | 1 | 1.010 |

Abbreviations: GVIF = generalized variance-inflation factor, df = degree of freedom.

**Supplemental table 14. Generalized variance-inflation factors of adjusted logistic regression models for diarrhea.**

| **Variables** | **Diarrhea** | | |
| --- | --- | --- | --- |
|  | **GVIF** | **df** | **GVIF^(1/(2*df))** |
| Age | 1.122 | 3 | 1.019 |
| Gender | 1.036 | 1 | 1.018 |
| Potentially inappropriate medication | 1.730 | 3 | 1.095 |
| Polypharmacy | 2.625 | 2 | 1.272 |
| Systolic blood pressure | 1.196 | 1 | 1.093 |
| Diastolic blood pressure | 1.130 | 1 | 1.063 |
| Body mass index | 1.022 | 1 | 1.011 |
| Fasting plasma glucose | 1.407 | 1 | 1.185 |
| 2-hour postprandial blood glucose | 1.415 | 1 | 1.189 |
| Glycated hemoglobin | 1.135 | 1 | 1.065 |
| Diabetic peripheral disease | 1.147 | 1 | 1.071 |
| Diabetic kidney disease | 1.374 | 1 | 1.172 |
| Diabetic retinal | 1.098 | 1 | 1.047 |
| Chronic respiratory disease | 1.139 | 1 | 1.067 |
| Osteoarthritis and rheumatoid arthritis | 1.132 | 1 | 1.064 |
| Chronic liver disease | 1.067 | 1 | 1.033 |
| Hypertension | 1.304 | 1 | 1.142 |
| Hyperlipemia | 1.317 | 1 | 1.147 |
| Cerebrovascular disease | 1.157 | 1 | 1.075 |
| Chronic kidney disease | 1.388 | 1 | 1.178 |
| Chronic gastrointestinal disease | 1.070 | 1 | 1.034 |
| Cardiovascular disease | 1.338 | 1 | 1.156 |
| Tumor | 1.038 | 1 | 1.019 |

Abbreviations: GVIF = generalized variance-inflation factor, df = degree of freedom.

**Supplemental table 15. Prevalence of PIMs by category, drug class and medications of older adults with diabetes cohort.**

| **PIM category** | **Drug class (percentage)** | **Medication (n)** |
| --- | --- | --- |
| **Category I: medications should be avoided by most older adults.** (73.64%) | | |
| Anticholinergics | First-generation antihistamines (11.65%) | Chlorpheniramine (620), Cyproheptadine (77), Diphenhydramine (329), Promethazine (457), Triprolidine (51) |
|  | Antiparkinsonian agents (0.15%) | Trihexyphenidyl (20) |
|  | Antispasmodics (2.76%) | Atropine (excludes ophthalmic) (40), Belladonna alkaloids (323) |
| Antithrombotics | Antithrombotics (0.10%) | Dipyridamole (13) |
| Anti-infective | Anti-infective (0.08%) | Nitrofurantoin (10) |
| Cardiovascular | Peripheral alpha-1 blockers (1.16%) | Doxazosin (114), Prazosin (27), Terazosin (12) |
|  | Digoxin (0.79%) | Digoxin (104) |
|  | Nifedipine (1.47%) | Nifedipine, immediate release (193) |
|  | Amiodarone (0.72%) | Amiodarone (95) |
| Central nervous system | Antidepressants (0.35%) | Amitriptyline (15), Clomipramine (1), Paroxetine (30) |
|  | Antipsychotics (1.21%) | Perphenazine (3), Chlorpromazine (3), Penfluridol, Sulpiride (7), Tiapride (3), Haloperidol (3), Amisulpride, Aripiprazole (4), Clozapine (11), Olanzapine (81), Paliperidone (2), Quetiapine (28), Risperidone (14), Ziprasidone (1) |
|  | Barbiturates (0.43%) | Phenobarbital (57) |
|  | Benzodiazepines (13.63%) | Alprazolam (385), Estazolam (1114), Lorazepam (25), Oxazepam (14), Clonazepam (75), Diazepam (183) |
|  | Nonbenzodiazepine, benzodiazepine receptor agonist hypnotics (1.15%) | Eszopiclone (17), Zolpidem (128), Zaleplon (6) |
| Endocrine | Androgens (0.03%) | Testosterone (4) |
|  | Desiccated thyroid (1.94%) | Desiccated thyroid (256) |
|  | Estrogens with or without progestins (0.01%) | Estrogens with or without progestins (1) |
|  | Insulin, sliding scale (5.09%) | Insulin, sliding scale (670) |
|  | Megestrol (0.08%) | Megestrol (11) |
|  | Sulfonylureas, long acting (5.86%) | Glimepiride (752), Glyburide (20) |
| Gastrointestinal | Metoclopramide (1.48%) | Metoclopramide (195) |
| Pain medications | Meperidine (0.49%) | Meperidine (65) |
|  | Non–cyclooxygenase-selective NSAIDs (2.26%) | Diclofenac (1406), Ibuprofen (1026), Ketoprofen (49), Meloxicam (44), Nabumetone (81), Naproxen (1), Piroxicam (71) |
|  | Indomethacin (1.68%) | Indomethacin (221) |
|  | Skeletal muscle relaxants (0.99%) | Chlorzoxazone (131) |
| Genitourinary | Desmopressin (0.02%) | Desmopressin (2) |
| **Category II: PIMs which should be avoided by older adults due to drug-disease or drug-syndrome interactions.** (2.10%) | | |
| Cardiovascular | Heart failure (0.32%) | NSAIDS (31), COX-2 inhibitors (1), Thiazolidinediones  (pioglitazone, rosiglitazone) (10) |
| Central nervous system | Dementia or cognitive impairment (0.53%) | Anticholinergics (7), Benzodiazepine (32), Nonbenzodiazepine, Benzodiazepine receptor agonist Hypnotics (4), Antipsychotics (27) |
|  | History of falls or fractures (1.18%) | Antiepileptics (26), Antipsychotics (8), Benzodiazepine (89), Nonbenzodiazepine, Benzodiazepine receptor agonist Hypnotics (14), TCAs (1), SSRIs (12), SNRIs (1), Opioids (4) |
|  | Parkinson disease (0.07%) | Antiemetics (5), Antipsychotics (4) |
| **Category III: Medications to be used with caution in older adults.** (24.26%) | | |
|  | Dabigatran (0.08%) | Dabigatran (10) |
|  | Rivaroxaban (0.08%) | Rivaroxaban (11) |
|  | Antipsychotics (1.02%) | Antipsychotics (135) |
|  | Carbamazepine (0.46%) | Carbamazepine (60) |
|  | Diuretics (15.04%) | Spironolactone (348), Indapamide (326), Torasemide (31), Furosemide (375), Hydrochlorothiazide (901) |
|  | Mirtazapine (0.26%) | Mirtazapine (34) |
|  | Oxcarbazepine (0.22%) | Oxcarbazepine (29) |
|  | SNRIs (0.09%) | Duloxetine (12) |
|  | SSRIs (1.44%) | Citalopram (50), Fluvoxamine (2), Fluoxetine (59), Paroxetine (30), Sertraline (49) |
|  | TCAs (0.12%) | Amitriptyline (15), Clomipramine (1) |
|  | Tramadol (1.17%) | Tramadol (154) |
|  | Dextromethorphan/quinidine (4.28%) | Dextromethorphan/quinidine (564) |

Abbreviations: PIMs = potentially inappropriate medications, n = number, NSAIDs = nonsteroidal anti-inflammatory drugs, COX-2 = cyclooxygenase-2, SNRI = serotonin-norepinephrine reuptake inhibitor, SSRI = selective serotonin reuptake inhibitor, TCA = tricyclic antidepressant.
